# Retraction: A new drilling method—Earthworm-like vibration drilling

**DOI:** 10.1371/journal.pone.0270081

**Published:** 2022-06-13

**Authors:** Peng Wang, Hongjian Ni, Ruihe Wang

Following the publication of this article [1], errors were identified in the selection of model calculation parameters and the programming solution process. Specifically, the internal tension force *T*_*t*_ was miscalculated as the displacement of different time was used incorrectly (Eqs. 19–20).

This error impacts the calculation of the axial force of the drill-string, and the results presented in Figs 4–11. Following re-analysis of these results with the errors corrected, the main results are no longer upheld.

In light of this issue, the authors retract the article. All authors agree with the retraction and apologize for the issues with the published article.
